# Intraocular Pressure Reduction after Femtolaser Assisted Cataract Surgery and Its Association with the Use of Ultrasound

**DOI:** 10.3390/medicina57050437

**Published:** 2021-05-01

**Authors:** Hana Abouzeid, Walter Ferrini, Murielle Bochud

**Affiliations:** 1 Department of Ophthalmology, Geneva University Hospitals, 1205 Geneva, Switzerland; 2 Clinical Eye Research Centre Adolph de Rothschild, 1208 Geneva, Switzerland; 3 Hôpital de la Providence, 2000 Neuchâtel, Switzerland; walter.ferrini@gmail.com; 4 Unisanté, Centre Universitaire de Médecine Générale et Santé Publique, University of Lausanne, 1011 Lausanne, Switzerland; murielle.bochud@unisante.ch

**Keywords:** intraocular pressure, femtolaser assisted cataract surgery, cataract surgery, femotcataract surgery, ultrasound

## Abstract

*Background and Objectives*: To quantify the change in intraocular pressure (IOP) after phacoemulsification in patients having undergone femtolaser assisted cataract surgery (FLACS), and study the influence of the use of ultrasound on this change. Setting: Jules-Gonin Eye Hospital, University Department of Ophthalmology, Lausanne, Switzerland. *Materials and Methods*: Interventional study. Methods: All consecutive cases operated with FLACS and with complete data for the studied parameters were selected for inclusion in the study. Data had been prospectively collected and was analysed retrospectively. Linear regression was performed to explore the association of change in IOP with time of measure, ultrasound use, sex, age, and duration of surgery. *Results*: There was a mean decrease in intraocular pressure of 2.5 mmHg (CI 95% −3.6; −1.4, *p* < 0.001) postoperatively. No association between the change in intraocular pressure and ultrasound time or effective phaco time was observed when the data were analyzed one at a time or in a multiple linear regression model. There was no association with sex, age, nuclear density, presence of pseudoexfoliation, duration of surgery, and time of ocular pressure measurement. Eyes with preoperative IOP ≥ 21 mmHg had a more significant IOP reduction after surgery (*p* < 0.0001) as did eyes with an anterior chamber depth <2.5 mm (*p* = 0.01). *Conclusion*: There was a decrease in intraocular pressure six months after FLACS in our study similar to that in the published literature for standard phacoemulsification. The use of ultrasound may not influence the size of the decrease, whereas the preoperative IOP and anterior chamber depth do. FLACS may be as valuable as standard phacoemulsification for cases where IOP reduction is needed postoperatively.

## 1. Introduction

Intraocular pressure (IOP) has been shown to increase after extracapsular cataract extraction (ECCE) in some patients [1], and decrease after phacoemulsification in others [2]. Furthermore, after ECCE, the expected mean IOP reduction is less significant than after phacoemulsification [2,3]. The reason for this is not known, but it has been suggested that the use of ultrasound during phacoemulsification may explain this difference in IOP reduction [4]. Indeed, phacoemulsification ultrasound has been shown to induce a potentially IOP-lowering stress response in normal trabecular meshwork culture cells through the IL-1, NF-kB, ELAM1 pathway. The use of ultrasound and the difference in pressure gradients that it induces activate the latter pathway. By increasing IL-1 production, this pathway then enhances the aqueous outflow through the trabecular meshwork [4]. 

Introduced in 2008 [5], femtolaser assisted cataract surgery (FLACS) is a promising new technological advance; it plays an ever-increasing role in cataract surgery, with proven advantages over manual surgery with regard to astigmatism, capsulotomy, and use of ultrasound [6]. In two prospective studies, of which one was randomized, FLACS allowed reduced use of ultrasound in a statistically significant way as compared to standard phacoemulsification, with a protective effect on endothelial cell loss, positively correlated with the reduction of effective phaco time (EPT) in eyes with comparable nuclear lens density [7,8].

We hypothesized that the ultrasound reduction in FLACS as compared to standard phacoemulsification parallels IOP changes. To address this hypothesis, we quantified and correlated IOP change with ultrasound delivery duration during FLACS, excluding major confounding factors. 

## 2. Materials and Methods

This study received approval of the Ethical Committee, Canton de Vaud, Lausanne, Switzerland (protocol 97/14Lausanne), and followed the tenets of the Declaration of Helsinki. 

### 2.1. Patients and Eyes

We reviewed the files of the 141 initial consecutive eyes that underwent FLACS at the Jules-Gonin Eye Hospital using the LenSx system (Alcon LenSx, Fort Worth, TX, USA) followed by phacoemulsification and insertion of an intraocular lens (IOL). Data were prospectively collected for all cases. All surgeries were performed at the Jules-Gonin Eye Hospital by the same surgeon (H.A.) between January 2013 and December 2013. Exclusion criteria included small pupils (<5.5 mm), previous corneal surgery, previous ocular trauma, nystagmus, poor eyelid opening, and glaucoma. Only files of eyes with the 6 month follow-up visit performed at the Jules-Gonin Eye Hospital and with complete data for the studied parameters were included in this study. The studied parameters include preoperative IOP (≥ and <21 mmHg), change between preoperative and 6 month follow-up IOP, preoperative and 6 month follow-up time of IOP measure, EPT, ultrasound (US) time, nuclear cataract lens density, age at surgery, sex, duration of surgery, presence of pseudoexfoliation, and preoperative anterior chamber depth (ACD; ≥ and <2.5 mm). The duration of the surgery was defined by the beginning of the FLACS procedure and the end of the manual procedure. The nuclear cataract lens density was assessed preoperatively by the single surgeon (H.A) on a 4 grade basis, with grade 4 being the hardest density.

### 2.2. Preoperative and Postoperative Examinations

All patients underwent a full ophthalmic evaluation before surgery, including slit-lamp biomicroscopy, Goldmann tonometry, and measurement of uncorrected and corrected distance and near visual acuity. Investigations included measurement of ACD and axial length (IOL Master 500, Carl Zeiss, Oberkochen, Germany) as well as anterior segment examination. The same examination was repeated after 6 months for patients followed at the Jules-Gonin Eye Hospital.

### 2.3. Surgical Procedure

All patients were instructed to instill topical nepafenac four times on the day preceding the surgery and one time on the day of the procedure. After admission to the hospital, all patients underwent topical anesthesia with tetracaine 1% and pupil dilation with an insert containing 0.28 mg tropicamide and 5.4 mg phenylephrine chlorhydrate (Mydriasert, Spectrum Thea Pharma SA, Schaffhausen, Switzerland) placed in the lower conjunctival sac.

The femtosecond laser procedure and the phacoemulsification surgery were performed in the same operating room. For the laser procedure, a lid speculum was placed, and the eye was disinfected with a topical solution of chlorhexidine gluconate 0.05% (Hibidil, CPS Cito Pharma Services GmbH, Uster, Switzerland,). The femtolaser procedure was performed using the LenSx femtolaser machine (Alcon-LenSx, Inc., Fort Worth, TX, USA). Preprogrammed surgeon templates were used for anterior capsulotomy of 4.9 mm (5 µJ pulse energy with a 350 µm delta up and delta down), lens fragmentation patterns (posterior capsule safety zone of 800 µm), and corneal primary and secondary incisions. The first 41 eyes underwent a chop pattern of lens fragmentation in 3 planes. The following eyes underwent an additional lens cylinder pattern (3 cylinders, 3.2 mm diameter). The 2.2 mm primary corneal incision (at 120°) was constructed on 3 planes and under 100% of the posterior depth. The secondary uniplanar corneal incisions were constructed with an angulation of 30 degrees. 

After completion of the laser procedure, all patients were instilled with a drop of unidose of phenylephrine 10%, tropicamide 0.5%, and diclofenac 1 mg. For the manual procedure, all patients were moved under the microscope for cataract extraction and insertion of an IOL after sterile surgical preparation. 

The laser-created corneal incisions were opened with a blunt spatula, and a viscoelastic device (Viscoat, Alcon, Inc., Fort Worth, TX, USA) was injected in the anterior chamber before the removal of the capsulotomy flap utilizing a forceps. The anterior chamber was decompressed before hydrodissection, which was performed with a slow and titrate technique as described by Roberts et al. Then, the anterior chamber was refilled with a viscoelastic device (Viscoat, Alcon, Inc., Fort Worth, TX, USA) for quadrants full segmentation with the Crozafon^®^ Prechopper (Janach, Como, Italy). The surgery was completed by phacoemulsification using the Infiniti Vision System Unit (Alcon, Inc., Fort Worth, TX, USA). A Kelmann tip with an inner diameter of 0.9 mm and an angulation of 30 degrees at the opening was used. The phacoemulsification settings used for quadrants emulsification were phaco power max 30% with Ozil pulse and 100% maximal torsional amplitude, bottle height 110 cm, maximum vacuum 350 mmHg, and maximum aspiration rate 35 cc/min. For removal of the remaining cortex, bimanual irrigation/aspiration through nasal and temporal incisions were performed. All IOLs were folded and implanted in the capsular bag using an injection cartridge through the corneal wound. All incisions were left sutureless, and corneal hydration was used if needed. 

### 2.4. Statistical Analysis

We used mean and standard deviation to describe continuous variables and percentage for categorical variables. We used mixed linear regression to explore factors associated with the difference in intraocular pressure (preoperative and 6 months postoperative) in order to account for the fact that some patients had two eyes operated on. We used a t-test to compare the difference in intraocular pressure between baseline and 6 months in selected strata. We used a cut-off *p*-value of 0.05 to declare statistical significance. Statistical analyses were conducted using Stata 13.0 (StataCorp LP, College Station, TX, USA). 

## 3. Results

The patient demographics and baseline characteristics are presented in Table 1. There was a mean decrease in intraocular pressure of 2.5mmHg (CI 95% −3.6; −1.4, *p* < 0.001) between the preoperative measurement and the measurement at 6 months. Mean (SD) US time was 23.2 (11.1) sec, and mean (SD) effective phaco time was 11.3 (8.0) sec. Table 2 presents the results of the one-at-a-time linear regression analysis of the change in intraocular pressure between the preoperative measurement and the measurement at 6 months in relation to the following studied parameters: time at which the IOP was measured, preoperative effective phaco time and ultrasound time, sex, and age. Table 3 presents the results of the multiple linear regression analysis of the change in intraocular pressure in relation to the same parameters. No association between the change in intraocular pressure and the presented parameters was observed when analyzed one at a time or in a multiple linear regression model. No association with the presence of pseudoexfoliation and the duration of surgery was found. 

In the group of eyes with preoperative IOP ≥ 21 mmHg, the 6 month mean IOP reduction was 8.75 mmHg (*N* = 5) and was 1.78 mmHg in the group with preoperative IOP < 21 mmHg (*N* = 64); this difference between the two groups was statistically significant (*p* < 0.0001). 

In the group of eyes with preoperative anterior chamber depth ≥ 2.5 mm (*N* = 21), the 6 month mean IOP reduction was 1.14 mmHg (SD = 0.45) and was 3.80 mmHg (SD = 1.02) in the group with preoperative anterior chamber depth <2.5mm (*N* = 17); this difference between the two groups was statistically significant (*p* = 0.01).

## 4. Discussion

FLACS is a recent technology still under evaluation with regard to its advantages and drawbacks [6,9]. Cataract surgery and the subsequent IOP lowering has become part of the glaucoma therapeutic arsenal [10,11]. In randomized controlled trials, IOP reduction was significantly higher after phacoemuslification than laser iridotomy for acute angle attack [12] and higher than phacotrabeculectomy for controlled [13] or uncontrolled primary angle closure [14]. In a large meta-analysis, lens extraction was shown to induce an IOP reduction of 2 to 4 mmHg [2]. Interestingly, with standard phacoemulsification or using different techniques such as manual small-incision cataract surgery, a higher preoperative IOP was associated with a larger postoperative reduction of the IOP [15,16,17] Whether IOP reduction depends on a specific surgical technique or whether it results from a deepening of the anterior chamber induced by lens extraction—particularly in angle-closure patients—has not been clarified [11,18]. The fact that no association has been found between the post-cataract-surgery deepening of the anterior chamber and the IOP reduction has weakened the latter hypothesis [19,20]. On the other hand, the fact that phacoemulsification induced a greater IOP decrease as compared to ECCE suggests a direct effect of ultrasound on the trabecular meshwork cells through an IL-1, NF-kB, ELAM1 pathway [4]. In this context, our results show that the IOP lowering effect of FLACS at 6 months post-surgery is comparable to that reported in published literature for standard phacoemulsification (2–4 mmHg) and is independent from the ultrasound time. Whether a minimal ultrasound threshold is sufficient to induce metabolic changes of the trabecular meshwork or whether ultrasound does not play a major role in IOP lowering remains to be explored. 

From a clinical point of view, similar IOP reduction can be expected after FLACS or standard phacoemulsification. Furthermore, we showed that postoperative IOP reduction was proportional to preoperative IOP, with preoperative IOP ≥ 21 mmHg showing a greater IOP reduction, in accordance with the published results of Poley et al. on standard cataract surgery [15,16] and of Shah et al. on FLACS 21. Glaucoma patients or ocular hypertensive patients may thus benefit from the IOP lowering effect of FLACS more than patients with low pressure. This is corroborated by the results of Shah et al. [21], which showed significantly greater IOP lowering after 6 months for a glaucoma/glaucoma suspect group who underwent FLACS as compared to a control group. In their study, there was no statistically significant correlation between the CDE and IOP change from baseline to 1 year, both in the glaucoma/glaucoma suspect group and the control group. 

In our study, we showed that patients with preoperative shallow anterior chamber might benefit more from the FLACS surgery in terms of IOP reduction. Nevertheless, the preoperative IOP rise during FLACS related to the suction procedure, the vacuum applied, and the optomechanical interface is a concern [22,23,24]. This IOP rise varies between 25 mmHg [20] and 36 mmHg [23,24] for the curved contact lens interface and can reach 90.8 mmHg with a 500 mmHg vacuum [24], but it appears to be much lower with a liquid optical immersion interface (16.6 mmHg, SD 1.8) [24]. This issue is a matter of constant improvement with the different FLACS machines, and the increase is already proven to be relatively low with the liquid interface [24]. In the near future, with the reduction of the suction-related IOP rise, FLACS may be recommended for lens-based glaucoma treatment just as standard phacoemulsification is currently. 

The weakness of the study is the lack of control group, the retrospective analysis, and the limited sample size. These observations thus require further confirmation by prospective controlled studies. 

## 5. Conclusions

In conclusion, in our study, FLACS induced a similar decrease in intraocular pressure at 6 months as in the published literature for standard phacoemulsification. FLACS reduced IOP after surgery regardless of the use of ultrasound, with a postoperative IOP reduction proportional to the preoperative IOP, and inversely proportional to the preoperative anterior chamber depth. FLACS may be as valuable as standard phacoemulsification for cases where IOP reduction is needed postoperatively. However, only randomly controlled trials of FLACS, performed with different machines and comparing the change in intraocular pressure in association with ultrasound parameters, could definitively confirm the present conclusions and make possible a precise comparison with standard phacoemulsification.

## Figures and Tables

**Table 1:** Baseline demographics and characteristics of the study group (*N* = 40).

Variable	*N*	Mean	Standard Deviation
Age (years)	40	72.9	8.3
Female gender, %	25	62.5	
Nuclear density, %			
1	0	0	
2	13	32	
3	25	63	
4	2	5	
Preoperative IOP (mmHg)	40	15.3	3.4
IOP at 6 months (mmHg)	40	16.4	5.9
Duration of surgery (min)	40	28.1	6.3
Effective phaco time (s)	40	11.3	8.0
US time (s)	40	23.2	11.1

**Table 2:** Results of the one-at-a-time linear regression analysis between the change in intraocular pressure (dependent variable) and the studied parameters.

Parameter	*N*	Regression Coefficient	95% Confidence Interval	*p*-Value
Time at IOP measure (h)	32	0.50	−0.09; 1.09	0.096
**Ultrasound use:**				
EPT	29	−0.54	−1.62; 0.54	0.33
US time	29	−0.01	−0.07; 0.10	0.78
Sex (female)	40	−1.66	−4.06; 0.75	0.18
Age	40	−0.11	−0.25; 0.03	0.13

IOP: intraocular pressure; EPT: effective phaco time; US: ultrasound.

**Table 3:** Results of the multiple linear regression analysis between the change between preoperative and 6 month intraocular pressure and the studied parameters (*N* = 22).

Parameters	*N*	Regression Coefficient	95% Confidence Interval	*p*-Value
Time at IOP measure	22	0.57	−0.21; 1.36	0.15
Ultrasound use:				
EPT	22	1.48	−2.06; 5.03	0.41
US time	22	−0.01	−016; 0.14	0.88
Sex (female)	22	−0.37	−4.93; 4.18	0.87
Age	22	−0.03	−0.35; 0.29	0.84

IOP: intraocular pressure; EPT: effective phaco time; US: ultrasound.

## Data Availability

Not applicable.

## What Was Known

- Phacoemulsification lowers intraocular pressure (IOP) postoperatively.
- The use of ultrasounds could play a role in this IOP reduction by inducing a change in the trabecular filtration rate.
- FLACS requires less ultrasound than standard phacoemulsification.

## What This Paper Adds

- FLACS induces a reduction of IOP after surgery.
- The size of the post FLACS IOP decrease is not related to the use of ultrasound.
- The post FLACS IOP reduction is proportional to the preoperative IOP, and inversely proportional to the preoperative anterior chamber depth.
- The IOP decrease after FLACS or phacoemulsification may not be different.
- Whether a minimal ultrasound threshold is sufficient to induce metabolic changes of the trabecular meshwork or whether ultrasound does not play a major role in IOP lowering remains to be explored.
